# Limited association between *Wolbachia* and *Plasmodium falciparum* infections in natural populations of the major malaria mosquito *Anopheles moucheti*

**DOI:** 10.1111/eva.13619

**Published:** 2023-11-27

**Authors:** Théo Mouillaud, Audric Berger, Marie Buysse, Nil Rahola, Josquin Daron, Jean‐Pierre Agbor, Sandrine N. Sango, Daniel E. Neafsey, Olivier Duron, Diego Ayala

**Affiliations:** ^1^ MIVEGEC, Univ. Montpellier, CNRS, IRD Montpellier France; ^2^ Montpellier Ecology and Evolution of Disease Network (MEEDiN) Montpellier France; ^3^ Faculté de Médecine et des Sciences Pharmaceutiques, Université de Douala Douala Cameroon; ^4^ Department of Immunology and Infectious Diseases Harvard T.H. Chan School of Public Health Boston Massachusetts USA; ^5^ Infectious Disease and Microbiome Program Broad Institute of MIT and Harvard Cambridge Massachusetts USA; ^6^ Medical Entomology Unit Institut Pasteur de Madagascar Antananarivo Madagascar

**Keywords:** *Anopheles moucheti*, malaria control, *Plasmodium* infection, *Wolbachia*

## Abstract

Since the discovery of natural malaria vector populations infected by the endosymbiont bacterium *Wolbachia*, a renewed interest has arisen for using this bacterium as an alternative for malaria control. Among naturally infected mosquitoes, *Anopheles moucheti*, a major malaria mosquito in Central Africa, exhibits one of the highest prevalences of *Wolbachia* infection. To better understand whether this maternally inherited bacterium could be used for malaria control, we investigated *Wolbachia* influence in *An. moucheti* populations naturally infected by the malaria parasite *Plasmodium falciparum*. To this end, we collected mosquitoes in a village from Cameroon, Central Africa, where this mosquito is the main malaria vector. We found that the prevalence of *Wolbachia* bacterium was almost fixed in the studied mosquito population, and was higher than previously recorded. We also quantified *Wolbachia* in whole mosquitoes and dissected abdomens, confirming that the bacterium is also elsewhere than in the abdomen, but at lower density. Finally, we analyzed the association of *Wolbachia* presence and density on *P. falciparum* infection. *Wolbachia* density was slightly higher in mosquitoes infected with the malaria parasite than in uninfected mosquitoes. However, we observed no correlation between the *P. falciparum* and *Wolbachia* densities. In conclusion, our study indicates that naturally occurring *Wolbachia* infection is not associated to *P. falciparum* development within *An. moucheti* mosquitoes.

## 1 INTRODUCTION

In the last decades, *Wolbachia* has emerged as a promising tool against vector‐borne diseases in addition to the classical control measures, such as the use of insecticides (Bourtzis et al., 2014; Caragata et al., 2021; Ferguson, 2018; Iturbe‐Ormaetxe et al., 2011; Ross et al., 2019). This endosymbiont bacterium presents two major characteristics that can be exploited as an alternative vector control strategy. First, *Wolbachia* bacteria can induce cytoplasmic incompatibility in their hosts to enhance their maternal transmission. Specifically, the host sperm‐egg compatibility is altered when infected males mate with uninfected females (Werren et al., 2008). Usually, this reproductive phenotype exhibits high prevalence in their hosts (Caragata et al., 2021; Ross et al., 2019). Therefore, releasing infected males in a non‐infected population should drastically reduce the vector density, called population suppression. This approach has been successfully employed to control dengue transmission by the mosquito *Aedes aegypti* (Crawford et al., 2020; Utarini et al., 2021). Second, this endosymbiont bacterium can mediate and protect their hosts from infection by a pathogen. This protective phenotype has been observed for a large variety of pathogens and hosts (Braquart‐Varnier et al., 2015; Cattel et al., 2016; Gupta et al., 2017; Martinez et al., 2014). Nevertheless, the underlying molecular mechanisms are still unknown. Some studies suggest a competition for the host cell resources that limits the parasite development (Paredes et al., 2016). However, other studies hypothesized the pre‐activation of the host innate immune system (Iturbe‐Ormaetxe et al., 2011; Pan et al., 2018). This implies that *Wolbachia* infection stimulates the host active defenses, and consequently has a protective effect against pathogens. This phenotype has been observed mainly when *Wolbachia* is artificially transferred into a new host (Ross et al., 2019). In natural systems, the prevalence of an endosymbiont with a protective phenotype infection can occur at intermediate or low frequencies if the intensity of reproductive manipulation is low (Hilgenboecker et al., 2008; Ross et al., 2019). The two phenotypes are not exclusive, and they have been concomitantly observed upon the release of *A. aegypti* infected with the *Wolbachia* strain *w*Mel (Ross et al., 2019, 2022).

In *Anopheles* mosquitoes, the discovery of *Wolbachia* infection in natural conditions has been controversial. For decades, it was thought that *Anopheles* vectors were not infected by this bacterium by multiple factors, including the presence of *Asaia* as antagonist to *Wolbachia* (Hughes, Dodson, et al., 2014; Hughes, Rivero, et al., 2014; Rossi et al., 2015). Few years ago, *Wolbachia* was detected in *Anopheles gambiae*, the major malaria vector in Africa (Baldini et al., 2014). However, other researchers have failed to detect *Wolbachia* in this species and raised doubts about this finding (Chrostek & Gerth, 2018; Sawadogo et al., 2022). *Wolbachia* was already studied in *Anopheles* by transfer of an exogenous strain into *An. gambiae* and *Anopheles stephensi* (Bian et al., 2013; Hughes et al., 2011; Kambris et al., 2010). Despite the promising effect on blocking malaria transmission, only one mosquito line of self‐sustainable infection by maternal inheritance has been established in *An. stephensi* (Bian et al., 2013; Hughes, Dodson, et al., 2014; Hughes, Rivero, et al., 2014), limiting its applicability. After the potential discovery of naturally occurring infected *An. gambiae* and *Anopheles coluzzii* populations, some authors investigated *Wolbachia* role in *Plasmodium falciparum* infection and revealed a strong negative correlation between *Wolbachia* presence and *P. falciparum* infection (Gomes et al., 2017; Shaw et al., 2016). Although most *Anopheles* species exhibit a low *Wolbachia* infection rate, between 0% and 20% (Ayala et al., 2019), few species show higher rates, particularly *Anopheles moucheti* (50%–70% in independent populations), in which vertical transmission was confirmed (Ayala et al., 2019; Walker et al., 2021). *Anopheles moucheti* sensu stricto (hereafter *An. moucheti*) is considered one of the major malaria vectors in Africa (Antonio‐Nkondjio et al., 2006; Fontenille & Simard, 2004). It belongs to a group of 3 species, among which *An. moucheti* is the main malaria vector (Antonio‐Nkondjio & Simard, 2013). This malaria mosquito is considered to be at the origin of human malaria from the transfer of the non‐human malaria parasites from primates to humans (Paupy et al., 2013). Moreover, it is broadly present in the forested areas of Central Africa (Ayala et al., 2009), where it contributes significantly to malaria transmission. Therefore, the high *Wolbachia* prevalence in this mosquito species offers a compelling opportunity to study how this endosymbiont bacterium modulates *Plasmodium* infection in its host. Unfortunately, one of the major drawbacks to experimental approaches is the absence of established *An. moucheti* laboratory colonies, limiting current investigations to mosquitoes sampled from natural populations.

In this study, we investigated the effect of *Wolbachia* on *P. falciparum* infection in a natural *An. moucheti* population in Cameroon, Central Africa. We screened mosquitoes to detect the presence of *Wolbachia* and *P. falciparum* using a newly developed qPCR assay. We further quantified the density of both microorganisms in *An. moucheti* specimens. With this approach, we empirically assessed the *Wolbachia* effect on *P. falciparum* infection in this mosquito under natural conditions. Our results contribute to the study of *Wolbachia* in *Anopheles* and its potential value to the development of new strategies for malaria control.

## 2 MATERIALS AND METHODS

### 2.1 Sample collection, mosquito identification, and DNA extraction

Mosquitoes were collected in Ndangueng, Cameroon, in February 2020 and July 2021. Adult females were sampled using the human landing catch method (HLC), following the recommendations of the National Ethical Committee in Cameroon (CNERSH N° 2020/07/1259/CE/CNERSH/SP). Mosquitoes were morphologically identified on the basis of taxonomical identification keys (Gillies & Coetzee, 1987; Gillies & De Meillon, 1968). Specimens belonging to the *An. moucheti* group were transported and kept alive for up to 10 days at the Malaria research Service of OCEAC, Yaoundé, Cameroon. For mosquitoes that died within the first 3 days after capture (gonotrophic cycle), abdomens were dissected and kept separately. All dead specimens were individually preserved in tubes containing a desiccant (silica gel, Sigma‐Aldrich) and stored at −20°C for molecular studies. DNA was extracted using the CTAB method, as described in Morlais et al. (2004). Briefly, samples were ground in 200 μL of 2% CTAB solution (1 M Tris HCl pH 8.0, 0.5 M EDTA, 1.4 M NaCl, 2% cetyltrimethylammonium bromide) and incubated at 65°C for 5 min. Total DNA was extracted in chloroform, precipitated in isopropanol, washed in 70% ethanol before resuspension in Qiagen TE buffer and storage at −20°C. Then, a sample subset was analyzed to identify members of the *An. moucheti* group using the species‐specific PCR assays developed by Kengne et al. (2007).

### 2.2 Wolbachia and *P. falciparum* detection and quantification

The detection and quantification of *Wolbachia* and *P. falciparum* in *An. moucheti* mosquitoes were assessed using quantitative PCR assays (here after qPCR). To this end, a plasmid carrying a specific fragment of gene from both targeted organisms was designed. All primers were designed with Primer3plus (Untergasser et al., 2012), targeting mono‐copy housekeeping genes to avoid over‐estimations: *An. moucheti GPDG* gene (F: 5′‐AAGTTGTTTCCGGACGTTTG‐3′; R: 5′‐CGTCGGATAGATTAATGGTG‐3′) (Nsango et al., 2023), *Wolbachia coxA* gene (GenBank accession number: MK755519.1) (F: 5′‐GGTGCTATAACTATGCTGCT‐3′; R: 5′‐TATGTAAACTTCTGGATGACC‐3′), and *P. falciparum Cox1* gene (GenBank accession number: LR605957.1) (F: 5′‐TTACATCAGGAATGTTATTGC‐3′; R: 5′‐ATATTGGATCTCCTGCAAAT‐3′) (Boissiere et al., 2013). To construct the plasmid, the three amplicons (*An. moucheti*, 111 bp; *Wolbachia*, 126 bp; *P. falciparum*, 120 bp) were cloned together (total length of 357 bp) (Appendix S1) in the pEX‐A128 vector. The primers, amplicons and plasmid were prepared by Eurofins.

The molecular quantification of *P. falciparum* and *Wolbachia* gene copies was carried out by qPCR. 1 μL of total DNA was added to a mixture (final volume of 10 μL) that contained 0.6 μL of each primer (see above) at 10 μM, 0.5 μL of 2X Mix SensiFast Sybr NO‐ROX Kit (Bioline), and 2.8 μL of sterile molecular biology grade water (Hyclone Hypure, Cytiva). Each plasmid contains a gene copy for each organism, therefore, calculating the number of plasmid copies, we can estimate the gene copies for both *Wolbachia* and *P. falciparum* in each mosquito. To construct the standard curve, the plasmid was diluted seven times from 10^−2^ to 10^−7^ (7.45 × 10^7^ to 7.45 × 10^2^ plasmid copies/μL). For each PCR plate, one separate mixture for each of the three targeted genes per sample and per plasmid dilution was prepared. In total, 24 samples were tested in each 96 PCR plate. Cycling conditions included an initial denaturation step at 95°C for 10 min, followed by 40 cycles of denaturation (95°C for 10 s), annealing (57°C for 5 s), and elongation (72°C for 20 s), followed by a melting step (95°C for 2 min, 68°C for 2 min, and then up to 97°C, holding for 15 s once at 97°C) with a continuous fluorescence detection to construct the melting curves, and a final cooling cycle at 40°C for 10 s. All qPCR assays were carried out on a LightCycler 480 (Roche Diagnostics) using the LightCycler 480 software version 1.5.1.

### 2.3 Statistical analysis and data visualization

Statistical analyses were carried out with R (R Core Team, 2022) and data modeling with the package “*tidyverse*” (Wickham et al., 2019). To estimate the repeatability of our quantitative measures, the mixed effects model framework implemented by the package “*rptR*” (Stoffel et al., 2020) was used with the “species” variable (i.e. *P. falciparum* or *Wolbachia*) as random‐effect predictors. Groups (i.e. infected vs. non‐infected mosquitoes) were compared with the Mann–Whitney test and the package “stats”. Correlation between ratios (i.e. *P. falciparum* and *Wolbachia*) were computed using the Pearson correlation coefficient (*r*) in R. Data, including all figures, were visualized using the package “*ggplot*” (Wickham, 2016) and associated packages, such as “*patchwork*” (Pedersen, 2020) and “*ggpubr*” (Kassambara & Kassambara, 2020).

## 3 RESULTS

### 3.1 Prevalence of malaria parasites and *Wolbachia* in *An. moucheti*

In total, we collected 2042 mosquitoes belonging to the *An. moucheti* group (Table S1). Although, *An. moucheti* is considered the predominant species of the group in this region (Ayala et al., 2009), we molecularly analyzed a subset of 179 mosquitoes to determine the species (Kengne et al., 2007) and attest that they were all assigned to the *An. moucheti* species. Therefore, we considered that *An. moucheti* was the only species of the group present in our sampling. To analyze their role in malaria transmission, the prevalence of *P. falciparum*‐infected mosquitoes was determined through qPCR assays. We found that 64/2042 females (3%) were infected with *P. falciparum*, a slightly higher rate than what previously reported (~1.4%) in the same geographic zone (Christophe Antonio‐Nkondjio et al., 2002). We then assessed *Wolbachia* presence in the infected mosquitoes and equal number of non‐infected to *Plasmodium*. In total, we analyzed 130 specimens. We removed specimens showing elevated cycle threshold (*c*_t_ > 26) to avoid any potential false positive amplification. Among the 113 specimens retained, 107 (95%) were infected by *Wolbachia*. This rate was higher than what previously observed in Gabon (71%; Ayala et al., 2019) and Cameroon (56.6%; Walker et al., 2021). We used these 113 mosquitoes for the subsequent analysis.

To estimate the reliability of our quantifications using the plasmid, we performed a repeatability analysis (Stoffel et al., 2020). We randomly re‐analyzed 21 mosquitoes from the 64 infected mosquitoes both organisms in this study. In our mixed effects model, we used the ratio of *Wolbachia* and *P. falciparum* value as explanatory variables, while the sample was used as random variable. The number of parametric bootstraps was 1000 for Gaussian data. The repeatability index (*R*) values were 0.853 for *Wolbachia* (*p* < 0.001) and 0.701 *P. falciparum* (*p* < 0.001).

### 3.2 Effect of *Wolbachia* on *P. falciparum* infection

**FIGURE 1:**
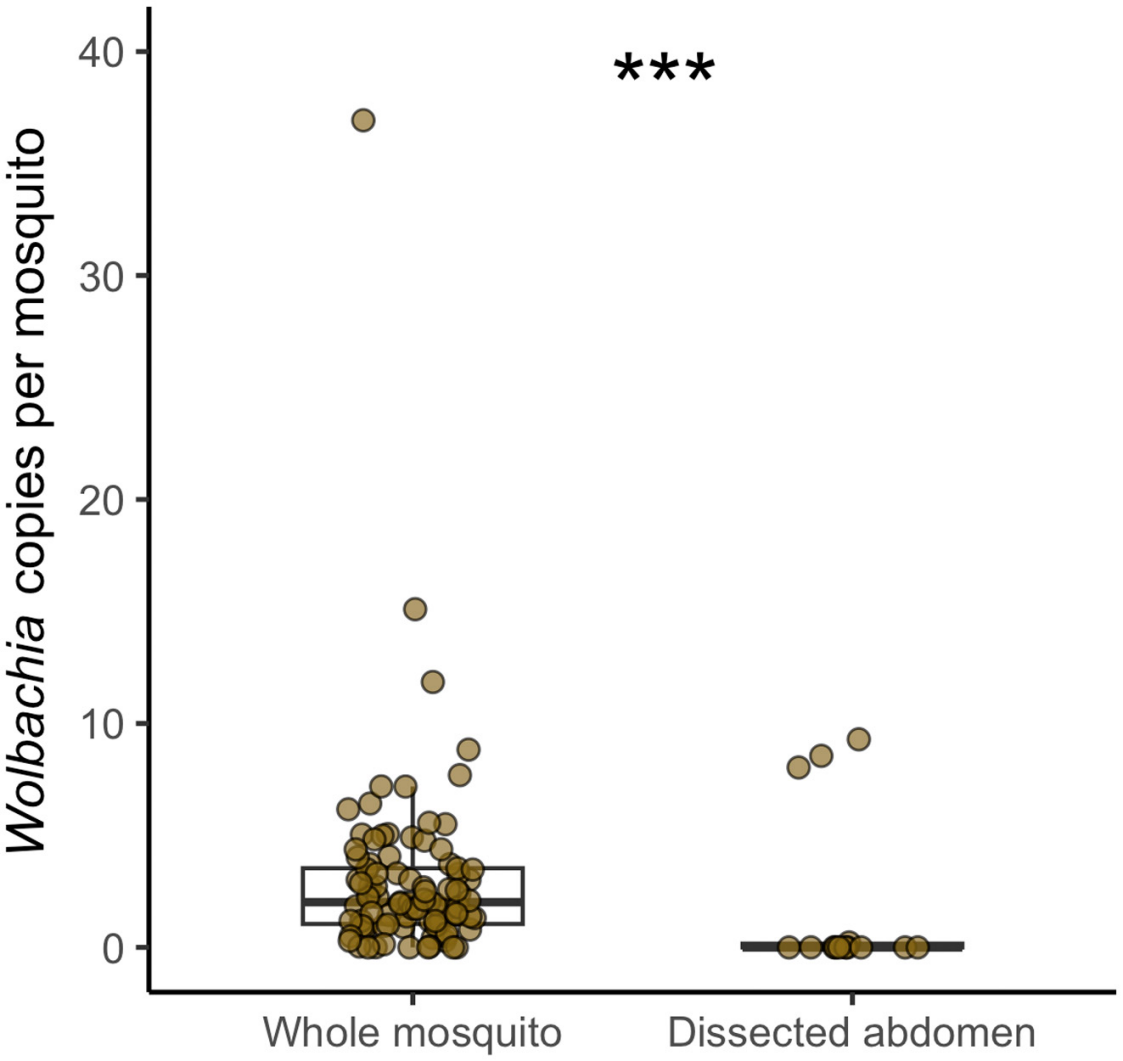
*Wolbachia* gene copies in *An. moucheti* (*n* = 93 whole mosquitoes; *n* = 14 dissected abdomens). ***p < 0.001 (Mann–Whitney).

Higher *Wolbachia* densities are usually observed in the ovaries of its hosts (Werren et al., 2008). To confirm that *Wolbachia* was present also in other *An. moucheti* tissues/organs, we compared the ratio of *Wolbachia coxA* gene copies to mosquito *GPDG* gene copies in whole mosquitoes (*n* = 93) and in abdomen alone (*n* = 14) (Figure 1). The ratio was significantly higher in whole mosquitoes (Mann–Whitney, *W* = 1387, *p* < 0.001). Specifically, the median ratios of *Wolbachia* gene copies to mosquito gene copies were 2.02 (maximum ratio = 36.93) in whole mosquitoes and 0.0006 (maximum ratio = 9.2) in dissected mosquitoes. When looking at *P. falciparum* infection, we found that only two dissected mosquitoes were infected. Conversely, the median ratio of *P. falciparum Cox1* gene copies to mosquito gene copies in whole mosquitoes was 0.17, with a maximum of 8.62, which was much lower than the ratio for *Wolbachia* gene copies.

**FIGURE 2:**
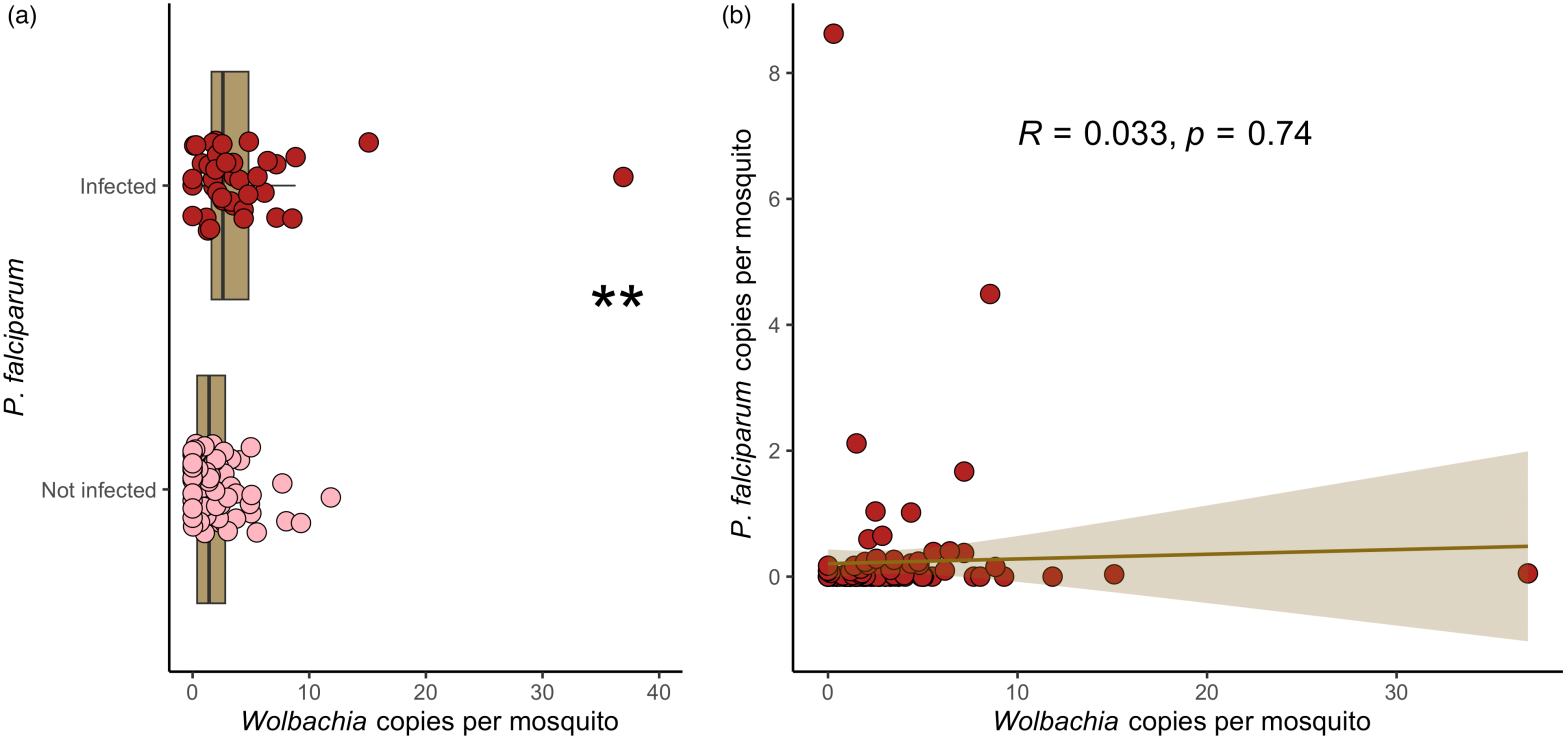
Association of *P. falciparum* and *Wolbachia* in *An. moucheti*. (a) Quantitative analysis of *P. falciparum* (infected vs. non‐infected) relative to *Wolbachia* gene copies (*n* = 38 *P. falciparum*‐infected specimens and *n* = 55 *P. falciparum* non‐infected specimens); **p = 0.004 (Mann–Whitney). (b) Correlation (Pearson) between the gene copies of *P. falciparum* and *Wolbachia* in *An. moucheti* (*n* = 113).

Prior studies suggested a potential role of *Wolbachia* in protecting *An. gambiae* from *P. falciparum* infection in natural populations (Gomes et al., 2017; Shaw et al., 2016). We first carried out a quantitative analysis to compare *Wolbachia* density in mosquitoes infected (*n* = 38) and uninfected with *P. falciparum* (*n* = 55) (Figure 2a). We found that *Wolbachia* density was slightly, but significantly higher in infected (mean gene copies = 4.21) than in uninfected (mean gene copies = 2.23) *An. moucheti* (Mann–Whitney, *W* = 996, *p* = 0.004). We then asked whether the *Wolbachia*‐*P. falciparum* relation was density‐dependent. Using the ratio of *Wolbachia* and *P. falciparum* copies relative to their host (*An. moucheti*), we observed a negative, but not significant correlation (Pearson, *R* = –0.11, *p* = 0.49, Figure 2b). These results suggest that *Wolbachia* does not hinder *P. falciparum* infection or development in this malaria vector, in agreement with its prominent role as major malaria vector in Central Africa (Fontenille & Simard, 2004).

## 4 DISCUSSION

*Wolbachia* is a maternally inherited endosymbiont proposed to reduce the malaria burden (Ross et al., 2019). In this study, we evaluated *Wolbachia* protective effect in *An. moucheti*, one of the major malaria vectors in Africa (Antonio‐Nkondjio et al., 2002; Fontenille & Simard, 2004). First, we estimated the number of mosquitoes infected with *Wolbachia* and *P. falciparum* under natural conditions. Second, we quantified *Wolbachia* and *P. falciparum* densities in *An. moucheti* and found that 3% (64/2042) of mosquitoes were infected by *P. falciparum* and 95% (107/113) by *Wolbachia*. We found that *Wolbachia* is not correlated with *P. falciparum* infection in *An. moucheti*. Unfortunately, we could not either control *Plasmodium* development, nor *Wolbachia* density in the mosquitoes, which could affect the observed phenotype (Gomes et al., 2017). Overall, this study allows better understanding *Wolbachia* place as a biological tool for malaria control in *An. moucheti*.

*An. moucheti* presents one of the highest *Wolbachia* prevalence rates in *Anopheles* (Ayala et al., 2019; Jeffries et al., 2018). *Wolbachia* is common across its geographical range (i.e. Cameroon, Gabon, Democratic Republic of Congo), ranging from more than 50% in Cameroon or Democratic Republic of Congo to almost fixed (95%). Differences in prevalence can be due to geographical heterogeneities, previously observed in other species (Ahmed et al., 2015), or technical issues. For instance, we used *coxA* instead of 16S for PCR‐based detection (Gomes et al., 2017; Jeffries et al., 2018). Nonetheless, our high prevalence is in agreement with results on maternal transmission in Gabon (from 90% to 100%) (Ayala et al., 2019), and with the theoretical prevalence of *Wolbachia* across its hosts (Zug & Hammerstein, 2012). We then quantified *Wolbachia* density in its mosquito host. We observed a median density of 2.02 gene copies of *Wolbachia* in *An. moucheti*. This number is similar to what observed in *Culex pipiens*, a mosquito with high infection rate (Berticat et al., 2002). Walker et al. (2021), also quantified *Wolbachia* density in *An. moucheti*. Unfortunately, they used a different method (i.e., *Wolbachia* 16S copies/ng DNA), making impossible the comparison between studies. Another study in *An. gambiae* proposed to normalize the host genome level using the S7 rRNA gene and two independent RT‐qPCR assays (Gomes et al., 2017), with similar results. We observed that *Wolbachia* densities varied greatly in our sample. The host age and the physiological status can greatly influence its density, as usually observed in other insect species (Duron et al., 2007; Tortosa et al., 2010; Unckless et al., 2009). Our *An. moucheti* mosquitoes were from a natural population, therefore, without control of age or physiological cycle, and this may explain the variation of *Wolbachia* densities we observed, which could affect *Plasmodium* infection (see below). Moreover, as mosquitoes were kept alive for at most 10 days, some of them could have developed eggs, possibly increasing *Wolbachia* density. All these factors need to be considered to accurate the association between *Wolbachia* and *Plasmodium* infection.

In Central Africa, *An. moucheti* plays a key role in malaria transmission (Christophe Antonio‐Nkondjio & Simard, 2013). Therefore, our first question was how this mosquito can be a major malaria vector despite this high *Wolbachia* prevalence if this endosymbiont exerts a negative impact on *P. falciparum* development as previously observed in *An. gambiae* (Gomes et al., 2017; Shaw et al., 2016). The *P. falciparum* infection rate (3%) in our *An. moucheti* population was relatively higher compared with other studies (Christophe Antonio‐Nkondjio et al., 2002). This could be explained by our experimental protocol: the probability of developing the infection would increase in mosquitoes that live longer (Dawes et al., 2009). Moreover, we quantified *P. falciparum* density in mosquitoes, and confirmed that in natural conditions, it is very low (Medley et al., 1993). When we studied *Wolbachia* effect on *P. falciparum* infection, we first quantitively analyzed *P. falciparum* and *Wolbachia* infection (Figure 2a). Our data suggests that *Wolbachia* density is not associated with malaria parasites development in this mosquito. Rather, our results revealed that *Wolbachia* density was higher in *P. falciparum‐*infected mosquitoes. Besides hindering *Plasmodium* infection, *Wolbachia* might also affect its development. Gomes et al. (2017), showed a significant association between *Wolbachia*, even at low density, with the malaria parasite in *An. gambiae* and *An. coluzzii*. This was particularly true in the artificially infected *An. coluzzii* colony with the development of sporozoites (Gomes et al., 2017). Unfortunately, we could not differentiate between sporozoite and oocyst infections. In our sampling, only one dissected mosquito (only sporozoites, Table S1) was positive for both *Wolbachia* and *Plasmodium* infection. On the other hand, our experimental design allowed the development of the parasite in the mosquito. Therefore, we could consider that an important proportion of mosquitoes could have produced sporozoites (Guissou et al., 2021). Moreover, only two *P. falciparum‐*positive mosquitoes were not infected with *Wolbachia*. Although this represents 33% of the 6 non‐*Wolbachia*‐infected mosquitoes (Table S1), it is much higher than the expected probability (0.15% expected vs. 33% observed) to find a non‐infected *Wolbachia* mosquito (5%) with *Plasmodium* parasites (3%) (expected probability = 0.05 × 0.03 = 0.0015). Unfortunately, due to the low number of uninfected *Wolbachia* specimens, we cannot exclude a random effect. Moreover, the *Plasmodium* density was similar between the non‐infected and infected *Wolbachia* mosquitoes (Figure 2a,b, Table S1). This interesting result need to be controlled with a larger sample size. In our study the *Wolbachia*‐*P. falciparum* density correlation was not significant (Figure 2b). In *An. gambiae*, two independent studies revealed a blocking role of *Wolbachia* in *P. falciparum* transmission in natural conditions (Gomes et al., 2017; Shaw et al., 2016). *Wolbachia* protective phenotype is very common when an exogenous *Wolbachia* strain invades a new host (Ross et al., 2019). It has been observed in *An. stephensi* and *An. gambiae*, where transfection of the *Wolbachia* strain (*wAlbB*) drastically reduced the ability to transmit the malaria parasites (Bian et al., 2013; Hughes et al., 2011). For instance, this phenotype has boosted the use of the *Wolbachia* strain *w*Mel, coming from the fruit fly, in *A. aegypti* (Schmidt et al., 2017; Utarini et al., 2021). On the other hand, in longtime established and high prevalence *Wolbachia*‐host associations, it is more unusual, and when it occurs, the bacterium prevalence is low, as it has been observed in the transmission of avian malaria parasites by *Culex* (Rivero & Gandon, 2018; Zele et al., 2014). Altogether, our results indicate that in *An. moucheti*, *Wolbachia* does not have a protective role against *P. falciparum* infection. These results are in agreement with its main malaria vector role in Central Africa. Although, future studies should study more carefully the *Wolbachia* density in salivary glands and the effect on sporozoite development.

Our finding may suggest that natural infections of *Wolbachia* are not useful for malaria control in *An. moucheti*, unlike what proposed by previous studies in *An. gambiae* (Ross & Hoffmann, 2021). Multiple factors could explain these discrepancies. First, while *An. gambiae* has been rarely found infected by *Wolbachia* with prevalence rates higher than 20%, *An. moucheti* exhibits one of the highest prevalence rates among the studied *Anopheles* species (Ayala et al., 2019; Jeffries et al., 2018). Therefore, we could hypothesize that *An. moucheti* immune system has been adapted to *Wolbachia* presence for long time as occur. Actually, the bacterium may increase the transmission in a long‐term association as it has been observed in the avian malaria system, where *Wolbachia*‐infected *Culex pipiens* mosquitoes transmit better than non‐infected (Hughes, Rivero, et al., 2014; Zele et al., 2014). Moreover, high *Wolbachia* prevalence is rather associated with cytoplasmic incompatibility than with protection against vector‐borne pathogens (Werren et al., 2008). Unfortunately, crossing experiments cannot be performed in *An. moucheti* because of the absence of laboratory colonies. The results of experiments using other *Anopheles* species with established laboratory colonies showed that colonies were infected with slightly different *Wolbachia* strains (Ayala et al., 2019; Jeffries et al., 2018; Walker et al., 2021), which could indicate an unidirectional CI (i.e., *Wolbachia*‐infected male mated with *Wolbachia* uninfected female), as evidenced in other systems (Werren et al., 2008). On the other hand, in hosts with low *Wolbachia* prevalence, this endosymbiont can be associated more with a protective phenotype (Oliver et al., 2010). This is the case in *An. gambiae* (Gomes et al., 2017). Despite the protective phenotype of the transinfected or naturally occurring *Wolbachia* strains against *Plasmodium* infection, currently, no program has been implemented in which *Wolbachia*‐infected *Anopheles* are used to reduce the malaria burden. The main challenge is to find a *Wolbachia* strain that can invade and exhibit a protective phenotype against malaria infection. The highly prevalent and *Anopheles*‐adapted *w*Anmo *Wolbachia* strain could represent an opportunity to carry out transfers to *Anopheles gambiae* cells, for instance. More studies controlling physiological parameters and parasite development on *An. moucheti* and *Wolbachia* are needed to determine the potential value of *Wolbachia* as a biological tool for malaria control.

## 5 CONCLUSIONS

The use of *Wolbachia* as vector control strategy is a reality. In the last decade, hundreds of thousands of *Wolbachia*‐infected *A. aegypti* specimens have been released in many different countries. As result, several dengue epidemics have been reduced and the wild vector population replaced by Wolbachia‐infected mosquito line (Utarini et al., 2021). In *Anopheles*, many studies have been conducted to determine *Wolbachia* place as a vector control strategy. Here, we characterized *Wolbachia* role in malaria transmission by *An. moucheti*, one of the major malaria vectors in Africa. Although, *Wolbachia* presence and density were not associated with protection against *P. falciparum* in *An. moucheti*, in agreement with its role in malaria transmission, this strategy could be proposed for other malaria vectors, such as *An. gambiae*.

## CONFLICT OF INTEREST STATEMENT

None declared.

## Supporting information


Table S1

Appendix S1

## Data Availability

Additional supporting information may be found online in the Supporting Information section at the end of the article.

## References

[eva13619-bib-0001] Ahmed, M. Z. , Araujo‐Jnr, E. V. , Welch, J. J. , & Kawahara, A. Y. (2015). *Wolbachia* in butterflies and moths: Geographic structure in infection frequency. Frontiers in Zoology, 12(1), 1–9. 26180539 10.1186/s12983-015-0107-z PMC4502936

[eva13619-bib-0002] Antonio‐Nkondjio, C. , Awono‐Ambene, P. , Toto, J.‐C. , Meunier, J.‐Y. , Zebaze‐Kemleu, S. , Nyambam, R. , Wondji, C. S. , Tchuinkam, T. , & Fontenille, D. (2002). High malaria transmission intensity in a village close to Yaounde, the capital city of Cameroon. Journal of Medical Entomology, 39(2), 350–355. 11931035 10.1603/0022-2585-39.2.350

[eva13619-bib-0003] Antonio‐Nkondjio, C. , Kerah, C. H. , Simard, F. , Awono‐Ambene, P. , Chouaibou, M. , Tchuinkam, T. , & Fontenille, D. (2006). Complexity of the malaria vectorial system in Cameroon: Contribution of secondary vectors to malaria transmission. Journal of Medical Entomology, 43(6), 1215–1221. 17162956 10.1603/0022-2585(2006)43[1215:cotmvs]2.0.co;2

[eva13619-bib-0004] Antonio‐Nkondjio, C. , & Simard, F. (2013). Highlights on *Anopheles nili* and *Anopheles moucheti*, malaria vectors in Africa. In S. Manguin (Ed.), Anopheles mosquitoes: New insights into malaria vectors [Internet]. InTech. 28045480

[eva13619-bib-0005] Ayala, D. , Akone‐Ella, O. , Rahola, N. , Kengne, P. , Ngangue, M. F. , Mezeme, F. , Makanga, B. K. , Nigg, M. , Costantini, C. , Simard, F. , Prugnolle, F. , Roche, B. , Duron, O. , Paupy, C. , & Paupy, C. (2019). Natural *Wolbachia* infections are common in the major malaria vectors in Central Africa. Evolutionary Applications, 12(8), 1583–1594. 10.1111/eva.12804 31462916 PMC6708434

[eva13619-bib-0006] Ayala, D. , Costantini, C. , Ose, K. , Kamdem, G. , Antonio‐Nkondjio, C. , Agbor, J.‐P. , Awono‐Ambene, P. , & Simard, F. (2009). Habitat suitability and ecological niche profile of major malaria vectors in Cameroon. Malaria Journal, 8(1), 307. 20028559 10.1186/1475-2875-8-307 PMC2805691

[eva13619-bib-0007] Baldini, F. , Segata, N. , Pompon, J. , Marcenac, P. , Shaw, W. R. , Dabire, R. K. , Levashina, E. A. , & Catteruccia, F. (2014). Evidence of natural *Wolbachia* infections in field populations of *Anopheles gambiae* . Nature Communications, 5, 3985. 10.1038/ncomms4985 PMC4059924 24905191

[eva13619-bib-0008] Berticat, C. , Rousset, F. , Raymond, M. , Berthomieu, A. , & Weill, M. (2002). High *Wolbachia* density in insecticide–resistant mosquitoes. Proceedings of the Royal Society of London Series B: Biological Sciences, 269(1498), 1413–1416. 10.1098/rspb.2002.2022 PMC1691032 12079666

[eva13619-bib-0009] Bian, G. , Joshi, D. , Dong, Y. , Lu, P. , Zhou, G. , Pan, X. , Xu, Y. , Dimopoulos, G. , & Xi, Z. (2013). *Wolbachia* invades *Anopheles stephensi* populations and induces refractoriness to *Plasmodium* infection. Science, 340(6133), 748–751. 10.1126/science.1236192 23661760

[eva13619-bib-0010] Boissiere, A. , Gimonneau, G. , Tchioffo, M. T. , Abate, L. , Bayibeki, A. , Awono‐Ambene, P. H. , Nsango, S. E. , & Morlais, I. (2013). Application of a qPCR assay in the investigation of susceptibility to malaria infection of the M and S molecular forms of *An. gambiaess* in Cameroon. PLoS One, 8(1), e54820. 23349974 10.1371/journal.pone.0054820 PMC3551906

[eva13619-bib-0011] Bourtzis, K. , Dobson, S. L. , Xi, Z. Y. , Rasgon, J. L. , Calvitti, M. , Moreira, L. A. , Bossin, H. C. , Moretti, R. , Baton, L. A. , Hughes, G. L. , Mavingui, P. , & Gilles, J. R. L. (2014). Harnessing mosquito‐*Wolbachia* symbiosis for vector and disease control. Acta Tropica, 132, S150–S163. 10.1016/j.actatropica.2013.11.004 24252486

[eva13619-bib-0012] Braquart‐Varnier, C. , Altinli, M. , Pigeault, R. , Chevalier, F. D. , Grève, P. , Bouchon, D. , & Sicard, M. (2015). The mutualistic side of *Wolbachia*–isopod interactions: *Wolbachia* mediated protection against pathogenic intracellular bacteria. Frontiers in Microbiology, 6, 1388. 26733946 10.3389/fmicb.2015.01388 PMC4679875

[eva13619-bib-0013] Caragata, E. P. , Dutra, H. L. , Sucupira, P. H. , Ferreira, A. G. , & Moreira, L. A. (2021). *Wolbachia* as translational science: Controlling mosquito‐borne pathogens. Trends in Parasitology, 37(12), 1050–1067. 34303627 10.1016/j.pt.2021.06.007

[eva13619-bib-0014] Cattel, J. , Martinez, J. , Jiggins, F. , Mouton, L. , & Gibert, P. (2016). *Wolbachia*‐mediated protection against viruses in the invasive pest *Drosophila suzukii* . Insect Molecular Biology, 25(5), 595–603. 27144810 10.1111/imb.12245

[eva13619-bib-0015] Chrostek, E. , & Gerth, M. (2018). Is *Anopheles gambiae* a natural host of *Wolbachia*? *bioRxiv* , 491449. 10.1101/491449 PMC6561020 31186318

[eva13619-bib-0016] Crawford, J. E. , Clarke, D. W. , Criswell, V. , Desnoyer, M. , Cornel, D. , Deegan, B. , Gong, K. , Hopkins, K. C. , Howell, P. , & Hyde, J. S. (2020). Efficient production of male *Wolbachia*‐infected *Aedes aegypti* mosquitoes enables large‐scale suppression of wild populations. Nature Biotechnology, 38(4), 482–492. 10.1038/s41587-020-0471-x 32265562

[eva13619-bib-0017] Dawes, E. J. , Churcher, T. S. , Zhuang, S. , Sinden, R. E. , & Basáñez, M.‐G. (2009). *Anopheles* mortality is both age‐and *Plasmodium*‐density dependent: Implications for malaria transmission. Malaria Journal, 8(1), 1–16. 19822012 10.1186/1475-2875-8-228 PMC2770541

[eva13619-bib-0018] Duron, O. , Fort, P. , & Weill, M. (2007). Influence of aging on cytoplasmic incompatibility, sperm modification and *Wolbachia* density in *Culex pipiens* mosquitoes. Heredity, 98(6), 368–374. 17519957 10.1038/sj.hdy.6800948

[eva13619-bib-0019] Ferguson, N. M. (2018). Challenges and opportunities in controlling mosquito‐borne infections. Nature, 559(7715), 490–497. 30046071 10.1038/s41586-018-0318-5

[eva13619-bib-0020] Fontenille, D. , & Simard, F. (2004). Unravelling complexities in human malaria transmission dynamics in Africa through a comprehensive knowledge of vector populations. Comparative Immunology, Microbiology and Infectious Diseases, 27(5), 357–375. 15225985 10.1016/j.cimid.2004.03.005

[eva13619-bib-0021] Gillies, M. T. , & Coetzee, M. (1987). A supplement to the Anophelinae of Africa south of the Sahara (p. 55). South African Institute for Medical Research.

[eva13619-bib-0022] Gillies, M. T. , & De Meillon, B. (1968). The Anophelinae of Africa south of the Sahara (Ethiopian zoogeographical region). Ethiopian Zoogeographical The Region.

[eva13619-bib-0023] Gomes, F. M. , Hixson, B. L. , Tyner, M. D. W. , Ramirez, J. L. , Canepa, G. E. , Silva, T. , Molina‐Cruz, A. , Keita, M. , Kane, F. , Traore, B. , Sogoba, N. , & Barillas‐Mury, C. (2017). Effect of naturally occurring *Wolbachia* in A*nopheles gambiae s.l*. mosquitoes from Mali on *Plasmodium falciparum* malaria transmission. Proceedings of the National Academy of Sciences of the United States of America, 114(47), 12566–12571. 10.1073/pnas.1716181114 29114059 PMC5703331

[eva13619-bib-0024] Guissou, E. , Waite, J. L. , Jones, M. , Bell, A. S. , Suh, E. , Yameogo, K. B. , Djègbè, N. , Da, D. F. , Hien, D. F. D. S. , Yerbanga, R. S. , Ouedraogo, A. G. , Dabiré, K. R. , Cohuet, A. , Thomas, M. B. , & Lefèvre, T. (2021). A non‐destructive sugar‐feeding assay for parasite detection and estimating the extrinsic incubation period of *Plasmodium falciparum* in individual mosquito vectors. Scientific Reports, 11(1), 9344. 10.1038/s41598-021-88659-w 33927245 PMC8085177

[eva13619-bib-0025] Gupta, V. , Vasanthakrishnan, R. B. , Siva‐Jothy, J. , Monteith, K. M. , Brown, S. P. , & Vale, P. F. (2017). The route of infection determines *Wolbachia* antibacterial protection in *Drosophila* . Proceedings of the Royal Society B: Biological Sciences, 284(1856), 20170809. 10.1098/rspb.2017.0809 PMC5474083 28592678

[eva13619-bib-0026] Hilgenboecker, K. , Hammerstein, P. , Schlattmann, P. , Telschow, A. , & Werren, J. H. (2008). How many species are infected with *Wolbachia*? – A statistical analysis of current data. FEMS Microbiology Letters, 281(2), 215–220. 18312577 10.1111/j.1574-6968.2008.01110.x PMC2327208

[eva13619-bib-0027] Hughes, G. L. , Dodson, B. L. , Johnson, R. M. , Murdock, C. C. , Tsujimoto, H. , Suzuki, Y. , Patt, A. A. , Cui, L. , Nossa, C. W. , & Barry, R. M. (2014). Native microbiome impedes vertical transmission of *Wolbachia* in *Anopheles mosquitoes* . Proceedings of the National Academy of Sciences of the United States of America, 111(34), 12498–12503. 25114252 10.1073/pnas.1408888111 PMC4151774

[eva13619-bib-0028] Hughes, G. L. , Koga, R. , Xue, P. , Fukatsu, T. , & Rasgon, J. L. (2011). *Wolbachia* infections are virulent and inhibit the human malaria parasite *Plasmodium falciparum* in *Anopheles gambiae* . PLoS Pathogens, 7(5), e1002043. 10.1371/journal.ppat.1002043 21625582 PMC3098226

[eva13619-bib-0029] Hughes, G. L. , Rivero, A. , & Rasgon, J. L. (2014). *Wolbachia* can enhance *Plasmodium* infection in mosquitoes: Implications for malaria control? PLoS Pathogens, 10(9), e1004182. 10.1371/journal.ppat.1004182 25187984 PMC4154766

[eva13619-bib-0030] Iturbe‐Ormaetxe, I. , Walker, T. , & Neill, S. L. O. (2011). *Wolbachia* and the biological control of mosquito‐borne disease. EMBO Reports, 12(6), 508–518. 10.1038/embor.2011.84 21546911 PMC3128286

[eva13619-bib-0031] Jeffries, C. L. , Lawrence, G. G. , Golovko, G. , Kristan, M. , Orsborne, J. , Spence, K. , Hurn, E. , Bandibabone, J. , Tantely, L. M. , Raharimalala, F. N. , Keita, K. , Camara, D. , Barry, Y. , Wat'senga, F. , Manzambi, E. Z. , Afrane, Y. A. , Mohammed, A. R. , Abeku, T. A. , Hedge, S. , … Walker, T. (2018). Novel *Wolbachia* strains in *Anopheles malaria* vectors from Sub‐Saharan Africa. Wellcome Open Res, 3, 113. 10.12688/wellcomeopenres.14765.2 30483601 PMC6234743

[eva13619-bib-0032] Kambris, Z. , Blagborough, A. M. , Pinto, S. B. , Blagrove, M. S. , Godfray, H. C. J. , Sinden, R. E. , & Sinkins, S. P. (2010). *Wolbachia* stimulates immune gene expression and inhibits *Plasmodium* development in *Anopheles gambiae* . PLoS Pathogens, 6(10), e1001143. 20949079 10.1371/journal.ppat.1001143 PMC2951381

[eva13619-bib-0033] Kassambara, A. , & Kassambara, M. A. (2020). Package ‘ggpubr’ . R package version 0.1, 6(0).

[eva13619-bib-0034] Kengne, P. , Antonio‐Nkondjio, C. , Awono‐Ambene, H. P. , Simard, F. , Awolola, T. S. , & Fontenille, D. (2007). Molecular differentiation of three closely related members of the mosquito species complex, *Anopheles moucheti*, by mitochondrial and ribosomal DNA polymorphism. Medical and Veterinary Entomology, 21(2), 177–182. 17550437 10.1111/j.1365-2915.2007.00681.x

[eva13619-bib-0035] Martinez, J. , Longdon, B. , Bauer, S. , Chan, Y.‐S. , Miller, W. J. , Bourtzis, K. , Teixeira, L. , & Jiggins, F. M. (2014). Symbionts commonly provide broad spectrum resistance to viruses in insects: a comparative analysis of *Wolbachia* strains. PLoS Pathogens, 10(9), e1004369. 25233341 10.1371/journal.ppat.1004369 PMC4169468

[eva13619-bib-0036] Medley, G. , Sinden, R. , Fleck, S. , Billingsley, P. , Tirawanchap, N. , & Rodriguez, M. (1993). Heterogeneity in patterns of malarial oocyst infections in the mosquito vector. Parasitology, 106(5), 441–449. 8341579 10.1017/s0031182000076721

[eva13619-bib-0037] Morlais, I. , Poncon, N. , Simard, F. , Cohuet, A. , & Fontenille, D. (2004). Intraspecific nucleotide variation in *Anopheles gambiae*: New insights into the biology of malaria vectors. The American Journal of Tropical Medicine and Hygiene, 71(6), 795–802. 15642974

[eva13619-bib-0038] Nsango, S. N. , Agbor, J.‐P. , Ayala, D. , Johnson, H. F. , Heaton, H. , Wagah, M. G. , Collins, J. C. , Krasheninnikova, K. , Pelan, S. E. , Pointon, D. B. , Sims, Y. , Torrance, J. W. , Tracey, A. , Uliano Da Silva, M. , Wood, J. M. D. , von Wyschetzki, K. , DNA Pipelines collective , McCarthy, S. A. , Neafsey, D. E. , … Lawniczak, M. (2023). A chromosomal reference genome sequence for the malaria mosquito, *Anopheles moucheti*, Evans, 1925 [version 1; peer review: 1 approved]. Wellcome Open Research, 8(507). 10.12688/wellcomeopenres.20259.1 PMC10690039 38046191

[eva13619-bib-0039] Oliver, K. M. , Degnan, P. H. , Burke, G. R. , & Moran, N. A. (2010). Facultative symbionts in aphids and the horizontal transfer of ecologically important traits. Annual Review of Entomology, 55, 247–266. 10.1146/annurev-ento-112408-085305 19728837

[eva13619-bib-0040] Pan, X. , Pike, A. , Joshi, D. , Bian, G. , McFadden, M. J. , Lu, P. , Liang, X. , Zhang, F. , Raikhel, A. S. , & Xi, Z. (2018). The bacterium *Wolbachia* exploits host innate immunity to establish a symbiotic relationship with the dengue vector mosquito *Aedes aegypti* . The ISME Journal, 12(1), 277–288. 29099491 10.1038/ismej.2017.174 PMC5739022

[eva13619-bib-0041] Paredes, J. C. , Herren, J. K. , Schüpfer, F. , & Lemaitre, B. (2016). The role of lipid competition for endosymbiont‐mediated protection against parasitoid wasps in *Drosophila* . mBio, 7(4), e01006‐16. 27406568 10.1128/mBio.01006-16 PMC4958261

[eva13619-bib-0042] Paupy, C. , Makanga, B. , Ollomo, B. , Rahola, N. , Durand, P. , Magnus, J. , Willaume, E. , Renaud, F. , Fontenille, D. , & Prugnolle, F. (2013). *Anopheles moucheti* and *Anopheles vinckei* are candidate vectors of ape *Plasmodium* parasites, including *Plasmodium praefalciparum* in Gabon. PLoS ONE, 8(2), e57294. 10.1371/journal.pone.0057294 23437363 PMC3577705

[eva13619-bib-0043] Pedersen, T. L. (2020). Patchwork: The composer of plots . R Package Version, 1(1), 182.

[eva13619-bib-0044] R Core Team . (2022). R: A language and environment for statistical computing. R Foundation for Statistical Computing. https://www.R‐project.org/

[eva13619-bib-0045] Rivero, A. , & Gandon, S. (2018). Evolutionary ecology of avian malaria: Past to present. Trends in Parasitology, 34(8), 712–726. 29937414 10.1016/j.pt.2018.06.002

[eva13619-bib-0046] Ross, P. A. , & Hoffmann, A. A. (2021). Vector control: Discovery of *Wolbachia* in malaria vectors. Current Biology, 31(11), R738–R740. 34102127 10.1016/j.cub.2021.04.038

[eva13619-bib-0047] Ross, P. A. , Robinson, K. L. , Yang, Q. , Callahan, A. G. , Schmidt, T. L. , Axford, J. K. , Coquilleau, M. P. , Staunton, K. M. , Townsend, M. , & Ritchie, S. A. (2022). A decade of stability for wMel *Wolbachia* in natural *Aedes aegypti* populations. PLoS Pathogens, 18(2), e1010256. 35196357 10.1371/journal.ppat.1010256 PMC8901071

[eva13619-bib-0048] Ross, P. A. , Turelli, M. , & Hoffmann, A. A. (2019). Evolutionary ecology of *Wolbachia* releases for disease control. Annual Review of Genetics, 53, 93–116. 10.1146/annurev-genet-112618-043609 PMC6944334 31505135

[eva13619-bib-0049] Rossi, P. , Ricci, I. , Cappelli, A. , Damiani, C. , Ulissi, U. , Mancini, M. V. , Valzano, M. , Capone, A. , Epis, S. , Crotti, E. , Chouaia, B. , Scuppa, P. , Joshi, D. , Xi, Z. Y. , Mandrioli, M. , Sacchi, L. , O'Neill, S. L. , & Favia, G. (2015). Mutual exclusion of *Asaia* and *Wolbachia* in the reproductive organs of mosquito vectors. Parasites & Vectors, 8, 278. 10.1186/s13071-015-0888-0 25981386 PMC4445530

[eva13619-bib-0050] Sawadogo, S. P. , Kabore, D. A. , Tibiri, E. B. , Hughes, A. , Gnankine, O. , Quek, S. , Diabaté, A. , Ranson, H. , Hughes, G. L. , & Dabiré, R. K. (2022). Lack of robust evidence for a *Wolbachia* infection in *Anopheles gambiae* from Burkina Faso. Medical and Veterinary Entomology, 36(3), 301–308. 35876244 10.1111/mve.12601 PMC10053554

[eva13619-bib-0051] Schmidt, T. L. , Barton, N. H. , Rašić, G. , Turley, A. P. , Montgomery, B. L. , Iturbe‐Ormaetxe, I. , Cook, P. E. , Ryan, P. A. , Ritchie, S. A. , Hoffmann, A. A. , O’Neill, S. L. , & Turelli, M. (2017). Local introduction and heterogeneous spatial spread of dengue‐suppressing *Wolbachia* through an urban population of *Aedes aegypti* . PLOS Biology, 15(5), e2001894. 10.1371/journal.pbio.2001894 28557993 PMC5448718

[eva13619-bib-0052] Shaw, W. R. , Marcenac, P. , Childs, L. M. , Buckee, C. O. , Baldini, F. , Sawadogo, S. P. , Dabiré, R. K. , Diabaté, A. , & Catteruccia, F. (2016). *Wolbachia* infections in natural *Anopheles* populations affect egg laying and negatively correlate with *Plasmodium* development. Nature Communications, 7. 10.1038/ncomms11772 PMC4895022 27243367

[eva13619-bib-0053] Stoffel, M. A. , Nakagawa, S. , & Schielzeth, H. (2020). An introduction to repeatability estimation with rpt R .

[eva13619-bib-0054] Tortosa, P. , Charlat, S. , Labbe, P. , Dehecq, J.‐S. , Barré, H. , & Weill, M. (2010). *Wolbachia* age‐sex‐specific density in *Aedes albopictus*: A host evolutionary response to cytoplasmic incompatibility? PLoS One, 5(3), e9700. 20300514 10.1371/journal.pone.0009700 PMC2838780

[eva13619-bib-0055] Unckless, R. L. , Boelio, L. M. , Herren, J. K. , & Jaenike, J. (2009). *Wolbachia* as populations within individual insects: Causes and consequences of density variation in natural populations. Proceedings of the Royal Society B: Biological Sciences, 276(1668), 2805–2811. 10.1098/rspb.2009.0287 PMC2839946 19419989

[eva13619-bib-0056] Untergasser, A. , Cutcutache, I. , Koressaar, T. , Ye, J. , Faircloth, B. C. , Remm, M. , & Rozen, S. G. (2012). Primer3—New capabilities and interfaces. Nucleic Acids Research, 40(15), e115. 22730293 10.1093/nar/gks596 PMC3424584

[eva13619-bib-0057] Utarini, A. , Indriani, C. , Ahmad, R. A. , Tantowijoyo, W. , Arguni, E. , Ansari, M. R. , Supriyati, E. , Wardana, D. S. , Meitika, Y. , & Ernesia, I. (2021). Efficacy of *Wolbachia*‐infected mosquito deployments for the control of dengue. New England Journal of Medicine, 384(23), 2177–2186. 34107180 10.1056/NEJMoa2030243 PMC8103655

[eva13619-bib-0058] Walker, T. , Quek, S. , Jeffries, C. L. , Bandibabone, J. , Dhokiya, V. , Bamou, R. , Kristan, M. , Messenger, L. A. , Gidley, A. , & Hornett, E. A. (2021). Stable high‐density and maternally inherited *Wolbachia* infections in *Anopheles moucheti* and *Anopheles demeilloni* mosquitoes. Current Biology, 31(11), 2310–2320.e2315. 33857432 10.1016/j.cub.2021.03.056 PMC8210651

[eva13619-bib-0059] Werren, J. H. , Baldo, L. , & Clark, M. E. (2008). *Wolbachia*: Master manipulators of invertebrate biology. Nature Reviews Microbiology, 6(10), 741–751. 10.1038/nrmicro1969 18794912

[eva13619-bib-0060] Wickham, H. (2016). Ggplot2: Elegant graphics for data analysis. Springer International Publishing.

[eva13619-bib-0061] Wickham, H. , Averick, M. , Bryan, J. , Chang, W. , McGowan, L. D. A. , François, R. , Grolemund, G. , Hayes, A. , Henry, L. , & Hester, J. (2019). Welcome to the Tidyverse. Journal of Open Source Software, 4(43), 1686.

[eva13619-bib-0062] Zele, F. , Nicot, A. , Berthomieu, A. , Weill, M. , Duron, O. , & Rivero, A. (2014). *Wolbachia* increases susceptibility to plasmodium infection in a natural system. Proceedings of the Royal Society B‐Biological Sciences, 281(1779), 20132837. 10.1098/rspb.2013.2837 PMC3924077 24500167

[eva13619-bib-0063] Zug, R. , & Hammerstein, P. (2012). Still a host of hosts for *Wolbachia*: Analysis of recent data suggests that 40% of terrestrial arthropod species are infected. PLoS One, 7(6), e38544. 10.1371/journal.pone.0038544 22685581 PMC3369835

