# Supplementary material for: Limited association between Wolbachia and Plasmodium falciparum infections in natural populations of the major malaria mosquito Anopheles moucheti
Source: Evol Appl. 2023 Nov 27;16(12):1999–2006. doi: 10.1111/eva.13619 (PMC10739076; doi:10.1111/eva.13619)
Supplement: Supplementary file 2 — Appendix S1 [file EVA-16-1999-s002.docx]

**Text S1.** Plasmid construction

AAGTTGTTTCCGGACGTTTGATCGTACGGCGTTACATTTGTTTTTAGAGTGCTATACTTACACTGTGTTCAAACAGCGCAGTTCCATCTATCACCATTAATCTATCCGACGTTACATCAGGAATGTTATTGCTAACACTACCGGTTTTAACTGGAGGAGTATTAATGTTATTATCAGACTTACATTTTAATACTTTATTTTTTGACCCAACATTTGCAGGAGATCCAATATGGTGCTATAACTATGCTGCTAACTGATCGCAATATTGGTACTTCCTTTTTTGATCCTGCTGGTGGCGGTGATCCTGTGTTATTTCAACACCTGTTTTGGTTTTTTGGTCATCCAGAAGTTTACATA
